# PlantSPEAD: a web resource towards comparatively analysing stress‐responsive expression of splicing‐related proteins in plant

**DOI:** 10.1111/pbi.13486

**Published:** 2020-10-25

**Authors:** Mo‐Xian Chen, Long‐Can Mei, Fan Wang, Iromi Kusum Wijethunge Boyagane Dewayalage, Jing‐Fang Yang, Lei Dai, Guang‐Fu Yang, Bei Gao, Chao‐Lin Cheng, Ying‐Gao Liu, Jianhua Zhang, Ge‐Fei Hao

**Affiliations:** ^1^ State Key Laboratory Breeding Base of Green Pesticide and Agricultural Bioengineering Key Laboratory of Green Pesticide and Agricultural Bioengineering Ministry of Education, Research and Development Center for Fine Chemicals Guizhou University Guiyang China; ^2^ CAS Key Laboratory of Quantitative Engineering Biology Shenzhen Institute of Synthetic Biology Shenzhen Institutes of Advanced Technology Chinese Academy of Sciences Shenzhen China; ^3^ Key Laboratory of Pesticide & Chemical Biology Ministry of Education College of Chemistry Central China Normal University Wuhan China; ^4^ Department of Biology Hong Kong Baptist University and State Key Laboratory of Agrobiotechnology The Chinese University of Hong Kong Shatin China; ^5^ State Key Laboratory of Crop Biology College of Life Science Shandong Agricultural University Taian China

**Keywords:** alternative splicing, splicing factor, stress, database, gene expression

Alternative splicing (AS) enhances transcriptome plasticity and proteome diversity in response to diverse growth and stress cues, which places AS at the crossroads of adaptation and environmental stress response (Chen *et al*., 2019a; Kuang *et al*., 2017). In recent years, the high‐throughput sequencing‐based analysis of plant transcriptomes has shown that this alternatively spliced mRNA processing is pervasive across plant species, with more than 80% and 70% of intron‐containing genes producing different isoforms in *Arabidopsis* and rice, respectively (Chen *et al*., 2019b; Zhu *et al*., 2017). In eukaryotic organisms, splicing is mediated by a large ribonucleoprotein (RNP) complex known as the spliceosome, which is comprised of a group of proteins called splicing‐related proteins. These proteins are important for recognizing splice sites or exonic splicing enhancers, and recent studies have revealed that the abnormal expression of SRPs is involved in stress tolerance in plants (Du *et al*., 2015; Gu *et al*., 2018). As upstream regulators, SRPs are believed to have a critical function in controlling the AS of stress‐responsive genes. Thus, it is important to shed light on the complex network of interactions that involves SRPs and their stress‐induced expression. However, this is complicated by the facts that some SRPs are still unknown, and available information is scattered in a large number of publications and various databases. Therefore, it would be useful to collect all the available information about plant SRPs together with their stress‐related expression characteristics into a unique resource, which would facilitate the discovery of new stress‐tolerant genes.

A number of AS‐related databases have been developed to facilitate the study of AS, including the SpliceAid series (i.e. series 1, 2 and F and (Piva *et al*., 2012), EuSplice (Bhasi *et al*., 2007) and SpliceInfo (Huang *et al*., 2004), etc. In particular, SpliceAid‐F provides structural information about splicing factors and their binding RNAs, whereas SpliceAid 2 includes the expression pattern of 62 human splicing proteins from 320 tissues and cell types and over 2200 target sites (Piva *et al*., 2012).

In this short communication, we present the plant splicing‐related protein expression and annotation database PlantSPEAD, an exhaustive collection of model plant SRPs, with data on their annotations, sequence information, functional domains, protein interaction partners and expression patterns in response to abiotic stresses, from a dozen existing databases and literature.

Specifically, we initially collected experimentally validated information on splicing‐related proteins in two model plant systems (i.e. *Arabidopsis thaliana* and *Oryza sativa*) by conducting a manually curated screening of the literature and databases. Subsequently, the strategy of reciprocal best hits performed by NCBI BLASTp program (*E*‐value < 10^−5^ and a minimum match degree >80%) was applied to detect potential orthologues of the aforementioned SRPs in 68 other plant species in the Phytozome database (v12.1). Finally, other information was manually extracted from public resources when available. This database provides a comprehensive view of the expression–function relationships of plant SRPs and their orthologues under stress, which may facilitate us identify new stress‐responsive plant SRPs. We hope that the knowledge base will advance our understanding of the biological functions of these plant SRPs in response to various environmental stimuli.

The obtained information on SRPs and analysis tools has been organized in PlantSPEAD and stored in the MySQL database (version 5.1.73), in a dedicated Linux server on a high‐performance computer cluster. The web interface is written in JavaScript using the React.js (i.e. for the input page, output page and data set visualization) and Ember.js (i.e., for the results and analysis pages) frameworks. Query and Chart functions were built for data search and visualization. Notably, this database was designed to provide comprehensive information for plant SRPs and facilitate the comparison of different SRPs in response to stress conditions (Figure 1a). Therefore, SRPs were organized into five categories, namely small nuclear ribonucleoproteins, splicing factors, splicing regulatory proteins, novel spliceosome proteins and possible splicing‐related proteins, which may help us sort out the genetics of each transcript isoform that is potentially functional under stress treatment. Moreover, the analysis of the expression of SRPs in shoot and root tissues under 9 abiotic stress treatments, namely cold, osmosis, salt, drought, genotoxicity, oxidation, UV‐B, wounding and heat, was performed.

**Figure 1:**
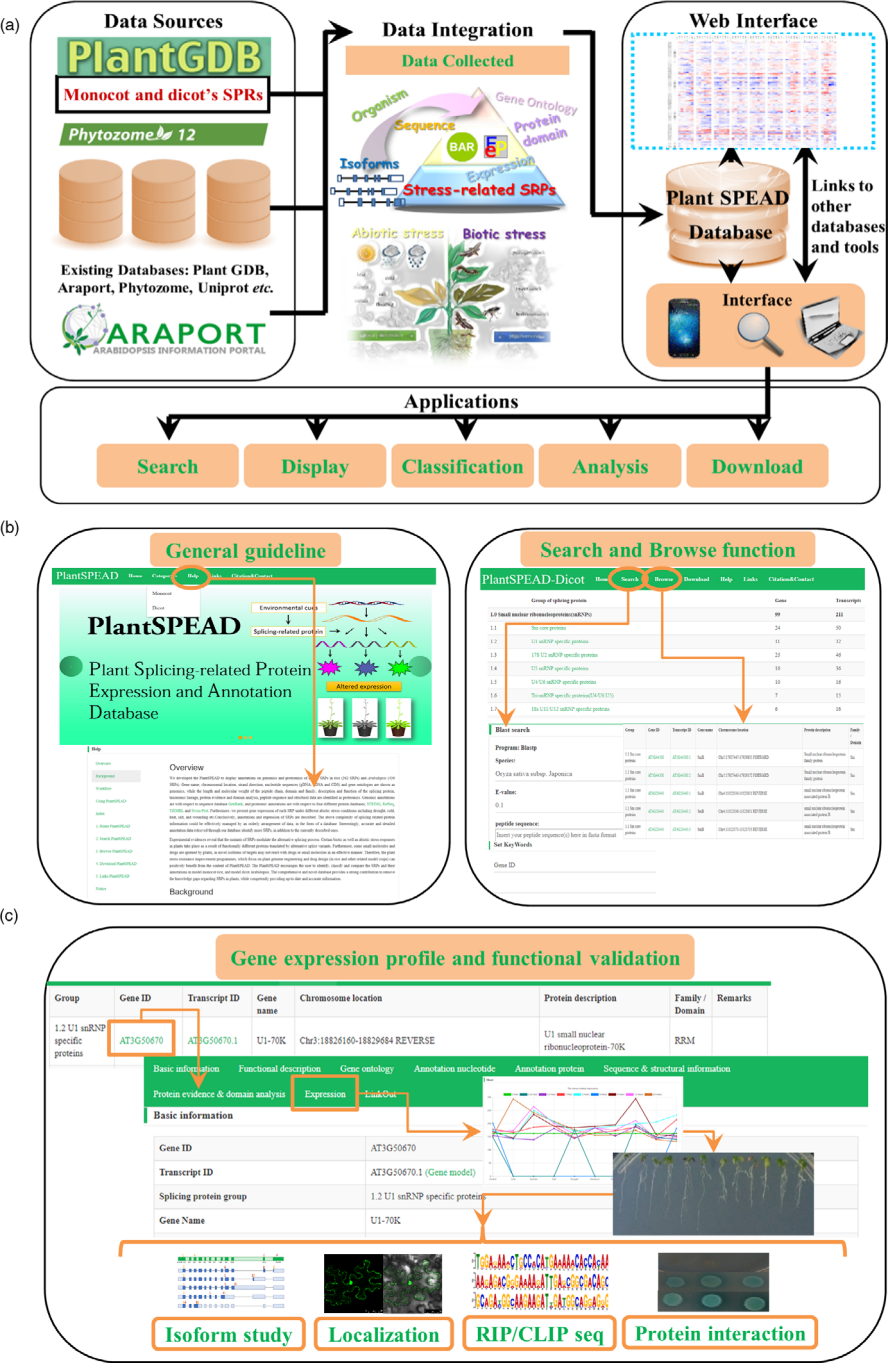
Structure, data and function of PlantSPEAD. (a) The data sources, data integration, web interface and potential applications of this database. (b) Illustration of the organization between the General guideline, Browse and Search pages in the PlantSPEAD web interface. (c) Example of how to locate a potential stress‐related plant SRP and of a follow‐up experimental design to study its biological function. [Colour figure can be viewed at wileyonlinelibrary.com]

PlantSPEAD contains information on dicotyledon and monocotyledon SRPs (including isoforms), organized in a relational database that is structured in several tables. In the main table, SRPs are listed together with data on the gene encoding the SRP, chromosome location, protein itself and family domain. Each SRP record in the main table is linked to records in other tables, hence gathering more data. We collected basic information, including the official symbols of genes, their synonyms and function, NCBI Gene IDs, and their links to NCBI HomoloGene. As for proteins, the PlantSPEAD database lists UniProt IDs, the type of RNA‐binding domains and domain analysis according to the PlantGDB, Araport11, Phytozome v12.1, UniProt, EMBL‐EBI and STRING databases. The expression data were extracted and re‐graphed using an eFP browser. All related information, including gene/protein annotation, sequence data and analysis tables, can be downloaded by users.

To date, we have collected the experimentally approved data of 43219 SRPs, 1,559 isoforms, 3,924 expression data and 6,236 annotations. There are 611 splicing isoforms from 362 SRP genes (219 to 7053 base pairs) and 948 splicing isoforms from 436 SRP genes (165 to 7080 base pairs) of model monocotyledonous and dicotyledonous plants, respectively. Overall, differentially expressed SRPs showed various induction or repression patterns in response to stress conditions. That is, 98 *Arabidopsis* and 68 rice genes were found to be differentially induced or repressed under stress treatments. Intriguingly, some *Arabidopsis* genes, such as At5g10800 and At2g32690, were consistently up‐regulated by various stresses, suggesting their potential roles in the response to diverse stress conditions.

The user‐friendly web interface of PlantSPEAD allows users to easily utilize and analyse all the data stored in the database (Figure 1b). For instance, a specific query interface was designed for PlantSPEAD, which facilitates dynamic queries by quick, advanced or BLAST searches (Figure 1b). Subsequently, the results are displayed in a table format, and users can activate an individual gene or isoform page by clicking on the hyperlinks on the corresponding gene or transcript ID. In order to find a stress‐specific plant SRP, users can locate a particular SRP using one of the three search approaches previously mentioned. By viewing the expression table, the user is able to find which particular stress treatment could activate or repress the expression of their gene of interest. Thereafter, the phenotypic characterization of this gene could be performed using mutants or overexpression transgenic plants under the same stress treatment as the one identified in the database. The step‐by‐step operation process and instructions for the use of the PlantSPEAD database can be found on the ‘Help’ webpage (Figure 1c).

To better describe the application of this web tool, we would like to use the stress‐related SRPs that were discovered in our laboratory as case examples. We successfully characterized several stress‐related SRPs using the aforementioned search criteria. An example with a plant U1 small nuclear ribonucleoprotein (U1‐snRNP) core protein, U1‐70K, is shown in Figure 1c. This core protein from the U1‐snRNP has been reported to play a pivotal role in 5' splice site determination. According to its expression analysis under stress treatments, we found that the transcription of this gene was induced after 6 h of osmotic stress. Thus, we tested the phenotypes of this SRP by using its T‐DNA insertional mutants. The results indicated that the mutant is insensitive to mannitol treatment, suggesting the involvement of the *Arabidopsis* U1‐70K gene in the response to osmotic stress (Chen *et al*., 2020).

In summary, we constructed a bioinformatics platform, namely PlantSPEAD, to provide a comprehensive repository of plant SRP classification, annotation, isoform collection and expression analysis. PlantSPEAD contains a flexible user interface to display all the information stored in the database. Furthermore, it includes the expression analysis data, guide document and reference links, making it a convenient and efficient bioinformatics platform for gene functional studies. Based on the information provided in this database, researchers can perform PCR analysis, design sequencing probes, carry out protein–protein interaction validations and evaluate the stress‐related phenotypes of these genes, among others. With the widespread interest in the functional study of plant SRPs, PlantSPEAD will be continuously updated, hence providing more valuable information for researchers. PlantSPEAD is freely available at http://chemyang.ccnu.edu.cn/ccb/database/PlantSPEAD or http://agroda.gzu.edu.cn:9999/ccb/database/PlantSPEAD.

## Conflict of interest

The authors declare no competing interests.

## Author contributions

G.F.H. designed the project. L.C.M., F.W., I.K.B.D., B.G., J.F.Y., C.L.C. and M.X.C. collected and analysed data. L.C.M. and F.W. built the web platform. M.X.C. and G.F.H. wrote the manuscript. L.D., Y.G.L., J.H.Z. and G.F.Y. critically reviewed and revised the manuscript.
